# Supplementary material for: GADF-VGG16 based fault diagnosis method for HVDC transmission lines
Source: PLoS One. 2022 Sep 23;17(9):e0274613. doi: 10.1371/journal.pone.0274613 (PMC9506613; doi:10.1371/journal.pone.0274613)
Supplement: S1 File — (DOCX) [file pone.0274613.s001.docx]

**YUPING**
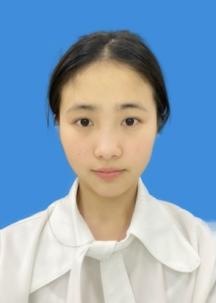
**YANG** (Non-member) She received the B.Sc. degree in electrical engineering and automation from Hunan University of Technology College of Science and Technology, China, in 2020 and is presently pursuing the M.Sc. degree in the intelligent control of electric power systems at Sichuan University of Science and Engineering, Zigong, China. Her research interests include power system protection and smart substations. Email: 2674670816@qq.com

**
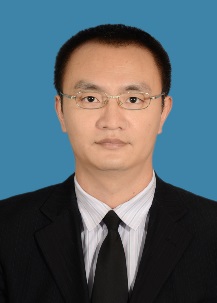
**HAO WU (Non-member) He received the Ph.D. degrees in the automation of electric power systems from Southwest Jiaotong University, Chengdu, China, in 2016, and is presently an Associate Professor with the School of Automation and Information Engineering, Sichuan University of Science & Engineering, Zigong, China. His research areas include power system protection and control, power systems simulation and modeling. Email: [wuhao801212@163.com](mailto:wuhao801212@163.com)

**
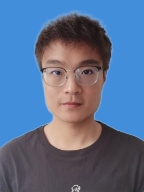
SIJING DENG** (Non-member) He received the B.Sc. degree in electrical engineering and automation from Sichuan University of Science and Engineering, China, in 2016, He research interests include fault diagnosis of active distribution network. Email: 873810537@qq.com

**QIAOMEI WANG**
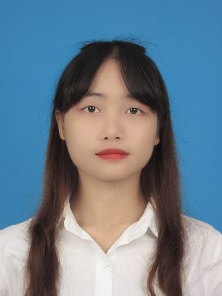
 (Non-member) She received the B.Sc. degree in electrical engineering and automation from Sichuan University of Science and Engineering, China, in 2018 and is presently pursuing the M.Sc. degree in the intelligent control of electric power systems at Sichuan University of Science and Engineering, Zigong, China. Her research interests include power system protection and smart substations. Email: wqm478712791@163.com

**
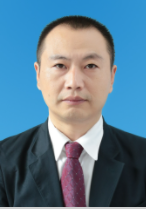
HONG** SONG (Non-member) he born in 1973, is a candidate for the academic leader of Sichuan Province, the head of the organization department of Sichuan University of Light Chemical Industry, and the head of the power system and its automation school-level discipline.

Mainly engaged in the research of smart grid, artificial intelligence technology and robotic technology, etc., has presided over or mainly researched "MOA arrester fault diagnosis expert system research", "research on intelligent distribution automation terminal", "transmission line intelligent diagnosis and monitoring analysis" More than 40 scientific research projects such as "Intelligent Diagnosis and Monitoring Analysis System of System Transmission Line", "Multi-sensor Fusion Technology Model and Application Research", "Development of Service Robot Control System Based on ARM and Embedded μC/OS-Ⅱ"; published academic papers More than 80 papers, more than 40 SCI, EI indexed.
