# Supplementary material for: GADF-VGG16 based fault diagnosis method for HVDC transmission lines
Source: PLoS One. 2022 Sep 23;17(9):e0274613. doi: 10.1371/journal.pone.0274613 (PMC9506613; doi:10.1371/journal.pone.0274613)
Supplement: Attachment — Submitted filename: Response to reviewers.docx [file pone.0274613.s007.docx]

Dear Editor,

The article "GADF-VGG16 based Fault diagnosis method for HVDC transmission lines " has been revised in strict accordance with the opinions of the peer reviewers. The specific changes are available for peer review.

**Reviewer #1:**

**Comment:**

1） The literature review is problematic. The review on technical development is insufficient.

2) The contributions should be simplified to highlight your work.
3) The authors spent too many pages introducing some well-known theories. It can be simplified.

4）The effectiveness of the proposed method needs to be verified by contrast experiments.
5) The comparison results with existing works should be proved by experiments rather than written description.

**An explanation of modification:**

1、In response to question 1)，The authors supplemented the literature review section as follows:

At present, experts and scholars have proposed a series of protection schemes for the protection of HVDC transmission lines. Among them, traveling wave protection, differential undervoltage protection is generally used as the main protection, longitudinal differential protection and low voltage protection as backup protection [4], while the traveling wave protection principle is most widely used in many protection methods. For example, in [5], based on the double-ended traveling wave method, it is proposed to use the time interval between the detection of the first incident traveling wave and the reflected wave at the detection point for the identification of faults inside and outside the zone. However, the method relies on the transmission of double-ended information, which has an impact on the quickness of the protection. Considering that traveling wave protection is susceptible to high resistance and fault distance, the author of [6] proposes to use traveling wave transmission principle combined with Teager energy operator to differentiate the internal fault from the external fault in the HVDC system. In this method, fault identification can be completed in a short time and the identification of fault types can be accurately achieved under high resistance faults at the remote end.However, how to accurately identify the traveling wave head in the traveling wave method is an insurmountable technical problem [7]. In [8],studies the internal and external faults identify method of HVDC transmission system based on distributed parameter model. This method does not rely on the traveling wave protection principle, and the accuracy of its fault detection depends on the setting of parameters of the transmission line.

In recent years, a large number of scholars have proposed the use of support vector machines (SVM), Back propagation neural networks（BP）, artificial neural networks (ANN), random forests and other methods [9-12] to study the problem of transmission line fault diagnosis.For example, Johnson et al [9] used the SVM classification mechanism to achieve fault identification and classification of HVDC transmission lines.The feature vector used in the classification modules is the standard deviation of the signals over half cycle before and after the occurrence of fault.However, the method does not fully exploit the waveform characteristics of the faulty traveling waves and the fault tolerance of the method needs to be further verified.

R. Kou et al [10] used the amount of electrical variation in transmission line fault conditions as a feature vector to train BP neural networks for fault diagnosis.This method has a good identification effect for in-zone faults, but does not consider the identification of out-of-zone faults.K. Moloi et al [11] used a particle swarm algorithm (POS) to optimize an artificial neural network (ANN), and then used the optimized ANN model to identify and classify different faults.The method can identify faults in and out of the zone with an accuracy of 99%. However, whether the method can also correctly identify fault types in high transition resistance and high noise environments remains to be proven.Wu et al [12] used random forest (RF) neural networks to achieve the selection of fault poles and identification of fault types on HVDC transmission lines. But, the extraction of fault features is more complex with this method.

Yet, the development of deep learning has brought new ideas to the field of transmission line fault diagnosis.The deep learning approach is able to learn deep abstract features in the data autonomously [13] and is suitable for complex transmission line fault diagnosis problems.Zhai et al [14] proposed to construct a fault diagnosis model for HVDC transmission lines using an improved convolutional neural network (CNN) to extract features and implement fault classification for current timing data.Compared with the traditional CNN network, the method has a certain improvement in recognition accuracy, but the CNN is not ideal for feature extraction of time-series signals.Therefore, it has been proposed to convert the time-series signal into a two-dimensional image, and then use CNN for fault feature extraction and classification.As Wang et al [15] proposed to convert one-dimensional time-series signals into two-dimensional grayscale images, and then use CNN for transmission line fault classification.However, the processing of the time-series signal into a grey-scale image in this method results in the loss of feature data.It can be seen that the application of deep learning to fault diagnosis on transmission lines is feasible and is subject to further in-depth research.

2、In response to question 2) and 3)，the author have simplified the paper,it can be seen in the revised manuscript.
3、In response to question 4）and 5），the author added the contrast experiment as follow:

It is compared with the published work related to intelligent fault diagnosis of HVDC transmission lines [9-14].In order to objectively verify the effectiveness of the method proposed in this paper, it is compared with the neural network models in the above intelligent fault diagnosis methods such as SVM, BP, ANN, RF, and CNN.The obtained test results are shown in Table 8.It can be seen from Table 8 that the model proposed in this paper has the highest recognition rate of transmission line faults among the six models.It can be seen that the model proposed in this paper has a better identification effect and can effectively solve the problem of transmission line fault identification.

**Table 8 Model recognition accuracy comparison**

| Neural network | training set accuracy/% | test set accuracy/% |
| --- | --- | --- |
| VGG16 | 99.01 | 100 |
| SVM | 92.84 | 92.55 |
| BP | 88.0 | 84.3 |
| ANN | 86.66 | 80.65 |
| RF | 98.55 | 98 |
| CNN | 88.46 | 92.08 |

**Reviewer #2:**

**Comment:**

1) Improve the writing style of the paper. Like, avoid writing like Reference [8] shows that....
   2) Improve the quality of the Images. It is hard to understand the meanings.
   3) Improve the results and discussion section by improving the comparison matrices like computational time and implications implementations.

4)Add sensitivity analysis with respect the data uncertainty.

**An explanation of modification:**

1、 In response to question 1）、2）：

The writing style of the paper and the images has been modified as required.

2、 In response to question 3):

In the results analysis section, the advantages of the proposed algorithm are comprehensively demonstrated from five aspects: data sensitivity analysis, ablation experiment analysis, algorithm performance analysis, comparison analysis of the recognition effect of different network structures, and comparison analysis with existing intelligent diagnosis algorithms.

1. Sensitivity analysis:In this section, the proposed technique performance is investigated through different fault characteristics applied along the HVDC Transmission line model presented in Fig.1. Experimental validation using the control variable method with different influencing factors such as different fault types, different transition resistances and different fault distances .It demonstrates that the fault diagnosis model proposed in this paper is not affected by the fault type. It also demonstrates that the model is less affected by the fault distance, and has a certain ability to withstand transition resistance.

②In the ablation experiment: In this section, the ablation experiments have verified that all the improvement points of this paper for VGG16 are effective.By the way, the FPS index is mentioned in Table 5 , that is the detection speed of the network. The improved VGG16 detection speed can detect 26 pictures per second, that is the algorithm proposed in this paper needs 0.038s to diagnose a fault.It meets the requirements of rapid mobility.

③Algorithm performance analysis: In this section, the performance of the fault diagnosis algorithm proposed in this paper is analyzed, and its anti-noise performance and anti-data loss performance are verified respectively. In addition, the proposed approach is robust to measurement noise and data loss errors.

④Comparative analysis of recognition effects of different network structures：In this section, We compare the network model proposed in this paper with the unimproved VGG16 network model as well as the LeNet and Alexnet network models。In comparison with lenet and alexnet, it can be seen that the deep network model VGG16 is better at recognition because the deep network model can better learn the deep abstract features of the fault data.Comparing the improved network model in this paper with the unimproved VGG16 model, it can be seen that the recognition accuracy of the improved VGG16 model is further improved.It shows that the GADF-VGG16 HVDC transmission line fault diagnosis model proposed in this paper can identify HVDC transmission line faults more accurately and efficiently.

⑤Comparison with other intelligent fault diagnosis methods:It is compared with the published work related to intelligent fault diagnosis of HVDC transmission lines [9-14].In order to objectively verify the effectiveness of the method proposed in this paper, it is compared with the neural network models in the above intelligent fault diagnosis methods such as SVM, BP, ANN, RF, and CNN.The result shows that the model proposed in this paper has the highest recognition rate of transmission line faults among the six models.It can be seen that the model proposed in this paper has a better identification effect and can effectively solve the problem of transmission line fault identification.

3、 In response to question 4):

Sensitivity analysis of data uncertainty can be found in Section 4.3.1 of this article.In this section, the proposed technique performance is investigated through different fault characteristics applied along the HVDC Transmission line model presented in Fig.1.The obtained results considering the effects of the different fault resistances,fault distances and types are shown in 1)、2)、3) , respectively.
