# Supplementary material for: GADF-VGG16 based fault diagnosis method for HVDC transmission lines
Source: PLoS One. 2022 Sep 23;17(9):e0274613. doi: 10.1371/journal.pone.0274613 (PMC9506613; doi:10.1371/journal.pone.0274613)
Supplement: Attachment — Submitted filename: Response to Reviewers.docx [file pone.0274613.s008.docx]

Dear Editor,

The article "GADF-VGG16 based Fault diagnosis method for HVDC transmission lines " has been revised in strict accordance with the opinions of the peer reviewers. The specific changes are available for peer review.

**Reviewer #1:**

**Comment 1:**

(1) The authors need to interpret the meanings of the variables.
(2) Please highlight your contributions in introduction.
(3) The abstract should be rewritten to reflect the significance of the proposed work. The current abstract shows a lot of background information.
(4) Conclusion: What are the advantages and disadvantages of this study compared to the existing studies in this area?
(5) The inspiration of your work must further be highlighted. Some suggested recent literatures should add.

**An explanation of modification:**

1、In response to question 1）：

The author adds corresponding explanations to the meanings of the variables involved in the article.The variables in the formula have been explained below the public announcement, and the variables in the table, such as EG, PG, etc., are explained in Section 1.1 of the article.

2、In response to question 2）：

This paper adopts the method of deep learning and proposes a fault diagnosis model for HVDC transmission based on the improved VGG16 network. The main contributions are as follows:

1）This paper uses VMD to decompose the fault voltage signal of the HVDC transmission line into modal components, and converts the selected IMF component into a color image through the Gramian Angular Difference Field (GADF), then input the images into the improved VGG16 for feature extraction and classification. The method uses a novel GADF encoding approach for data pre-processing, constructing bijective mappings in one-dimensional time series and two-dimensional spatial series, which will not cause the loss of feature information[16].

2）This paper improves the traditional VGG16 model structure by adding the BN layer and the convolution layer with the densely connected structure to accelerate the training and convergence of the network and realize the reuse and enhancement of features. At the same time, the global average pooling layer is used to replace the fully connected layer, which reduces the amount of model parameters and calculation time, making the proposed method more suitable for rapid fault diagnosis.

3）In this paper, the improved VGG16 model is applied to the fault diagnosis of HVDC transmission lines, and compared with other intelligent fault diagnosis methods, the results show that fault diagnosis algorithm based on GADF-VGG16 is more reliable and the fault identification accuracy is higher.

3、In response to question 3）：

The summary section has been modified as requested as follows：

Since HVDC transmission lines undertake the important task of electric energy transportation, in order to improve the reliability of power supply and maintain the safe and stable operation of the power system, the role of fault diagnosis of transmission lines is very important.In this paper,an intelligent fault detection scheme for high-voltage direct current (HVDC) transmission line based on the Gramian Angular Difference Field (GADF) and the improved convolutional neural network VGG16(VGG16) is proposed. This method first performs variational modal decomposition (VMD) on the original fault voltage signal, and then uses the correlation coefficient method to select the appropriate intrinsic mode function (IMF) component, and converts it into a two-dimensional image using the Gramian Angular Difference Field(GADF). Finally, the improved VGG16 network is used to extract and classify fault features adaptively to realize fault diagnosis. The result of the comparative experiment show that the model proposed in this paper can adaptively extract and classify fault features, has high fault detection accuracy, and has good anti-noise ability and fault tolerance.At the same time, it is proved that the GADF-VGG16 fault identification model can be effectively applied to the fault diagnosis of transmission lines.And it also provides a new idea for further applying deep learning to the research of transmission line fault diagnosis.

4、In response to question 4）：

Compared with the existing related research, the advantages of the method proposed in this paper are as follows:：First of all, the algorithm proposed in this paper breaks the traditional thinking, converts one-dimensional fault signals into two-dimensional images for adaptive extraction of fault features, and better combines the advantages of convolutional neural networks in image processing to realize intelligent fault diagnosis of HVDC transmission lines. Secondly, compared with other intelligent algorithms, the improved VGG16 fault identification model proposed in this paper has higher fault identification accuracy. At the same time, the algorithm proposed in this paper is less affected by noise, has strong anti-interference ability, and has strong learning ability and generalization performance.

The disadvantages of the method proposed in this paper are as follows：Limited to laboratory conditions, the fault diagnosis models proposed in this paper are all carried out in a simulation environment, and there is a lack of fault data in the actual operation process to verify the proposed model.

5、In response to question 5）：

To further highlight the source of inspiration for this paper, the author adds some recent literature such as [17] [22] [23] [25]. The idea of processing the original data can be seen from the literature [17], the idea of converting one-dimensional data into a two-dimensional image is mainly from the literature [22] [23], and finally the improvement idea of the VGG16 network mainly comes from Literature [25].

**Reviewer #3:**

**Comment :**

(6) Correct typological mistakes and mathematical errors：The authors improved their paper by adding important parts making their research results more clear to the readers. I appreciate that. The intelligent fault detection is a more and more the subject of the research the authors trying to find better solutions for the high-voltage direct current transmission system. By the responses to the reviewers the authors clarified all the parts of their research. In my opinion this is a good paper.

**An explanation of modification:**

I am very grateful to the reviewers for their recognition. The authors have made further revisions for the shortcomings of the article. I hope that the reviewers will criticize and correct the revised manuscript submitted by the authors.

**Journal Requirements:**

Please review your reference list to ensure that it is complete and correct. If you have cited papers that have been retracted, please include the rationale for doing so in the manuscript text, or remove these references and replace them with relevant current references. Any changes to the reference list should be mentioned in the rebuttal letter that accompanies your revised manuscript. If you need to cite a retracted article, indicate the article’s retracted status in the References list and also include a citation and full reference for the retraction notice.

**modification to references:**

Compared with the original manuscript, the added references are mainly to show some recent problems in the field of transmission line fault diagnosis research and some new research ideas. Added the following references:

[1] Li Zhenxing, Tan Hong, Ye Shiyun, Li Zhenhua, Xu Yanchun. Analysis of the research status of UHV DC line protection[J]. High-voltage electrical appliances,2018,54(05):184-189.DOI:10.13296/j.1001-1609.hva.2018.05.029.
[2] Hao, W., Mirsaeidi, S., Kang, X., Dong, X., & Tzelepis, D. (2018). A novel traveling-wave-based protection scheme for LCC-HVDC systems using Teager energy operator. International Journal of Electrical Power & Energy Systems, 99, 474–480.
[3] Muniappan, Mohan. (2021). A comprehensive review of DC fault protection methods in HVDC transmission systems. Protection and Control of Modern Power Systems. 6. 10.1186/s41601-020-00173-9.
[4] Wu H, Wang Q, Yu K, Hu X, Ran M (2020) A novel intelligent fault identification method based on random forests for HVDC transmission lines. PLoS ONE 15(3): e0230717. <https://doi.org/10.1371/journal.pone.0230717>
[5] ZHAO Zhongqiu, ZHENG Peng, XU Shoutao, et al. Object detection with deep learning: a review [ J]. IEEE T ransactions on Neural Networks and Learning Systems, 2019, 30(11) :3212-323
[6] Wang, J.; Zheng, X.D.; Tai, N.L. DC Fault Detection and Classification Approach of MMC-HVDC Based on Convolutional Neural Network. In Proceedings of the 2018 2nd IEEE Conference on Energy Internet and Energy System Integration (EI2), Beijing, China, 20–22 October 2018.
[7] Z. Wang, T. Oates. Encoding time series as images for visual inspection and classification using tiled convolutional neural networks[C ], Proceedings of the 2015 Association for the Advancement of Artificial Intelligence (AAAI) Workshops, pp.40- 46.
[8] Y. Shen, W. Zheng, W. Yin, A. Xu and H. Zhu, "Feature Extraction Algorithm Using a Correlation Coefficient Combined With the VMD and Its Application to the GPS and GRACE," in IEEE Access, vol. 9, pp. 17507-17519, 2021, doi: 10.1109/ACCESS.2021.3049118.
[9] Xiao Xiong, Xiao Yuxiong, Zhang Yongjun, Song Guoming, Zhang Fei.Application Research of Data Augmentation Method Based on Two-dimensional Grayscale Image in Motor Bearing Fault Diagnosis[J].Chinese Journal of Electrical Engineering,2021,41(02):738-749 .DOI: 10.13334/j.0258-8013.pcsee.200834.
[10] TONG yu, PANG xinyu, WEI zihan. Fault diagnosis method of rolling bearing based on GADF-CNN[J]. Vibration and shock, 2021,40(05):247-253+260.
[11] Yang, H., Ni, J., Gao, J. et al. A novel method for peanut variety identification and classification by Improved VGG16. Sci Rep 11, 15756 (2021). https://doi.org/10.1038/s41598-021-95240-y

At the same time, compared with the original manuscript, the author has deleted the content of the article, and the content of these references has been deleted, so the author deleted the corresponding references:

[1] Y. Lecun, L. Bottou, Y. Bengio and P. Haffner, "Gradient-based learning applied to document recognition," in Proceedings of the IEEE, vol. 86, no. 11, pp. 2278-2324, Nov. 1998, doi: 10.1109/5.726791.
[2] Krizhevsky A, Sutskever I, Hinton G E. Imagenet classification with deep convolutional neural networks[C]//Advances in neural information processing systems. 2012: 1097-1105.
[3] Simonyan K, Zisserman A. Very Deep Convolutional Networks for Large-Scale Image Recognition[J]. arXiv preprint arXiv:1409.1556, 2014
