# Supplementary material for: GADF-VGG16 based fault diagnosis method for HVDC transmission lines
Source: PLoS One. 2022 Sep 23;17(9):e0274613. doi: 10.1371/journal.pone.0274613 (PMC9506613; doi:10.1371/journal.pone.0274613)
Supplement: Attachment — Submitted filename: Response to Reviewers.docx [file pone.0274613.s009.docx]

Reviewer #1:

Comments:

（1）In my opinion the current version of the abstract is not suitable. It should clearly state the manuscript main contribution to the body of knowledge, and the motivation that lead the author to this research.
（2）How about the computation complexity of the proposed method.
（3）The contributions should be simplified to highlight your work.
（4）Conclusion: What are the advantages and disadvantages of this study compared to the existing studies in this area?
（5）The inspiration of your work must further be highlighted. Some suggested recent literatures should add according to previous comments.
（6）The paper is in need of revision in terms of eliminating grammatical errors, and improving clarity and readability.

An explanation of modification:

1、The reply to comment (1) is as follows：

The abstract part is described from three aspects, the first is the description of the importance of fault diagnosis of transmission lines and some current problems in this research. Then, the technical route of the method proposed in this paper is summarized. Finally, the advantages of the proposed method and its contribution to fault diagnosis research are obtained from the experimental results. The details are as follows:

Transmission lines are the most prone to faults in the power transmission system, and high-precision fault diagnosis is crucial for quick troubleshooting. In the current intelligent fault diagnosis research methods, there are problems such as difficulty in accurately extracting fault features, low fault identification accuracy and low fault tolerance. In order to solve these problems, this paper proposes an intelligent fault diagnosis method for high-voltage direct current transmission lines (HVDC) based on Gram angle field difference field (GADF) and improved convolutional neural network VGG16. This method first performs variational modal decomposition (VMD) on the original fault voltage signal, and then uses the correlation coefficient method to select the appropriate intrinsic mode function (IMF) component, and converts it into a two-dimensional image using the Gramian Angular Difference Field(GADF). Finally, the improved VGG16 network is used to extract and classify fault features adaptively to realize fault diagnosis. In order to improve the performance of the VGG16 fault diagnosis model, batch normalization, dense connection and global average pooling techniques are introduced. The comparative experimental results show that the model proposed in this paper can deeply mine fault features and has a high fault diagnosis accuracy. In addition, the method is not affected by fault type, transition resistance and fault distance, has good anti-interference ability, strong fault tolerance, and has high potential in practical applications

2、The reply to comment (2) is as follows：

Judging the pros and cons of an algorithm is mainly measured from two aspects: the execution time of the algorithm (time complexity) and the storage space (space complexity) that it needs to occupy

1) The time complexity of the VMD algorithm can be expressed as: $Time～O(n)$where n denotes the number of program executions, and in this paper n=k, k denotes the number of IMF components of the vmd decomposition.

2)The time complexity of the GADF algorithm can be expressed as: $Time～O(n)$, where n is the number of times the program is executed, and in this paper n is equal to the number of samples.

3)Time complexity of the convolutional neural network VGG16.

In a convolutional neural network, The computing time is mainly spent on the convolution operation and the operation of the fully connected layer, and the operation of the fully connected layer accounts for most of the time. The greater the number of operations, the greater the computational resources consumed and the longer the time spent. In this paper, in order to reduce the time complexity of VGG16, the global average pooling layer is used instead of the fully connected layer, which greatly reduces the amount of computation in the network, i.e. greatly reduces the time complexity of the network.

The time complexity of a single convolutional layer expressed as (11):

$Time～O(M^{2}\cdot k^{2}\cdot C_{in}\cdot C_{out})$ （11） $M=(X-K+2*Padding)/Stride+1$ （12）

Where M represents the size of the output feature map of each convolution kernel, and its expression is as in Equation (12)，which is determined by the four parameters of input matrix size X, convolution kernel size K, padding, and stride; In the Equation (11), the k denotes the edge length of each convolutional kernel ; *C_in_* denotes the number of channels of each convolutional kernel, also represents the number of input channels; and *C_out_* denotes the number of convolutional kernels , also represents the number of output channels. It can be seen that the time complexity of each convolutional layer is fully determined by the output feature area M^2^, the convolutional kernel area *k^2^*, and the number of input and output channels.

The overall time complexity of the VGG16 network can be expressed as:

$Time～O(\sum_{l=1}^{D} M_{l}^{2}\cdot k_{l}^{2}\cdot C_{l-1}\cdot C_{l}+\sum_{j=1}^{D_{FC}} C_{j-1}\cdot M_{j}^{2}\cdot m_{j})$ （13）

Where D denotes the number of all convolutional layers of the neural network,also represents the network depth; *l* denotes the first convolutional layer of the neural network; *C_l_* denotes the number of output channels *C_out_* of the lth convolutional layer of the neural network, also represents the number of convolutional kernels in that layer. *D_FC_* denotes the number of fully connected layers, *m_j_* denotes the number of features to be output in that layer.

In this paper, the global average pooling layer is used to replace the fully connected layer, so the overall time complexity of the VGG16 network proposed in this paper can be expressed as (14):

$Time～O(\sum_{l=1}^{D} M_{l}^{2}\cdot k_{l}^{2}\cdot C_{l-1}\cdot C_{l}+C_{j-1}\cdot m_{j})$ （14）

2) Space Complexity of convolutional neural network VGG16:

The space complexity is a measure of the size of the storage space temporarily occupied by an algorithm during the running process. which can be expressed as (15) :

$Space～O(\sum_{l=1}^{D} K_{l}^{2}\cdot C_{l-1}\cdot C_{l}+\sum_{l=1}^{D} M^{2}\cdot C_{l})$ （15）

In the ablation experiment, it can be seen that the improved VGG16 algorithm proposed in this paper has a network parameter size of 69MB after removing the fully connected layer. Compared with the unimproved VGG16, it can be seen that the memory occupied by the network parameters is reduced by 179MB. It can be seen that the improved VGG16 has greatly improved the space complexity.

3、The reply to comment (3) is as follows：

The author has refined the contribution of this paper, and summarized it from two aspects of data processing and network improvement, as follows:

(1) Based on the GADF algorithm, the one-dimensional fault signal is converted into a two-dimensional color image, aiming to use deep learning to mine deeper fault feature information.
(2) The batch normalization algorithm is introduced to effectively speed up network convergence and prevent overfitting. The dense connection method of the convolutional layer is designed to speed up the network training and convergence speed while realizing feature reuse and enhancement. Aiming at the slowing down of the calculation speed caused by the large amount of network parameters, a global average pooling algorithm is introduced, which effectively reduces the amount of network parameters.
(3) The fault diagnosis model of GADF-VGG16 is proposed , which improve the fault diagnosis accuracy, and the superiority of the proposed algorithm is verified by an example.
4、The reply to comment (4) is as follows：

The author revised the conclusion part, highlighting the advantages and disadvantages of the proposed algorithm compared with the existing research. The specific revisions are as follows:

Aiming at the problem that the existing fault diagnosis methods are difficult to accurately extract fault characteristics when faced with complex fault characteristics, this paper proposes a fault diagnosis method for HVDC transmission lines based on GADF-VGG16. The algorithm proposed in this paper breaks the traditional thinking, converts the one-dimensional fault signal into a two-dimensional image for self-adaptive extraction of deep fault features, and better combines the advantages of convolutional neural network in image processing to realize intelligent fault diagnosis of HVDC transmission lines. The simulation results show that the proposed algorithm has the following advantages:

（1）The method proposed in this paper uses GADF to convert one-dimensional fault data into two-dimensional color images, retains the temporal correlation in the data, and uses deep learning algorithms to achieve high-dimensional feature extraction. Compared with the method of converting the data into a two-dimensional grayscale image in the literature [18], the data processing method proposed in this paper will not cause the loss of characteristic data, so that the fault diagnosis model has a higher fault identification accuracy.
（2）In this paper, the VGG16 network is improved by adding BN layer and convolutional layer of densely connected structure to speed up the training and convergence speed of the network while realizing feature reuse and enhancement; at the same time, the global average pooling layer is used to replace the fully connected layer, reducing The amount of model parameters and computation time make the proposed method more suitable for rapid fault diagnosis.
（3）Compared with traditional intelligent algorithms, the fault diagnosis model based on improved VGG16 constructed in this paper can fully extract deep fault features. Compared with the fault diagnosis algorithm based on SVM, BP, RF and other shallow neural networks in literature [9-12], the algorithm proposed in this paper has higher fault recognition accuracy, and the experimental results show that it is not affected by transition resistance, fault type and fault. It has strong anti-interference ability and fault tolerance.

On the other hand, the method proposed in this paper also has some limitations. Due to the huge size of the actual HVDC transmission system, the fault situation of the transmission line is more complicated. Limited to laboratory conditions, the fault diagnosis models proposed in this paper are all carried out in a simulation environment, and there is a lack of fault data in the actual operation process to verify the proposed model.

In order to further improve the fault diagnosis model of HVDC transmission lines, how to verify the proposed diagnosis model in combination with the actual fault data of HVDC transmission lines is the focus of the next research work.

5、The reply to comment (5) is as follows：

In order to highlight the source of inspiration for this study, some recent literatures have been added to the introduction and main text. Based on previous research, the research ideas of this paper are obtained. The specific revisions are as follows:

As a research hotspot in recent years, deep learning has been widely used in image processing, target detection, fault diagnosis and other fields. As Jin et al. [13] introduced an intelligent fault diagnosis method for train axle box bearings based on parameter optimization of VMD and improved DBN. The method uses the gray wolf optimization algorithm (GWO) to optimize VMD and DBN, and solves the parameters of VMD. The problem is set, and VMD is used to decompose the signal, and the feature information of the modal component with the largest correlation coefficient is extracted through the multi-scale distribution entropy. Finally, DBN is used to identify the characteristic information to realize the weak fault diagnosis of bearings.Reference [14] proposed an intelligent diagnosis algorithm based on continuous wavelet transform and Gaussian convolution deep belief network. In this method, the one-dimensional vibration signal is converted into a two-dimensional image and the deep belief network is used for feature extraction, which avoids the artificial extraction of fault features. influences. Smart algorithms have achieved significant improvements over traditional algorithms.Reference [15] proposed a new method for intelligent fault diagnosis based on time-frequency images and deep learning. This method uses short-time Fourier transform to obtain time-frequency images, and combines the multi-sensory data fusion method of deep residual network to carry out the analysis of machine bearings. For fault diagnosis, compared with a single type of fault signal, this method can achieve better diagnostic accuracy.The development of deep learning has also brought new ideas to the field of transmission line fault diagnosis.Reference [16] proposes a fault diagnosis method for HVDC systems based on stacked sparse autoencoders, which utilizes stacked sparse autoencoders for automatic feature extraction, classification and identification. This method makes full use of the self-learning ability of the deep learning algorithm to realize the accurate identification of regional faults.Zhai et al [17] proposed to construct a fault diagnosis model for HVDC transmission lines using an improved convolutional neural network (CNN) to extract features and implement fault classification for current timing data.Compared with the traditional CNN network, the method has a certain improvement in recognition accuracy, but the CNN is not ideal for feature extraction of time-series signals.Therefore, drawing on the idea of converting time series signals into two-dimensional images and combining deep learning algorithms in the field of bearing fault diagnosis[14/15/24], Many scholars have begun to try to use this research method in fault diagnosis of HVDC transmission lines.For example, Wang Jun et al. [18] proposed to convert one-dimensional time series signals into two-dimensional grayscale images, and then use CNN to classify transmission line faults. However, the process of converting the time series signal into grayscale image in this method results in the loss of characteristic data. It can be seen that it is feasible to apply deep learning to fault diagnosis of transmission lines, and further in-depth research is needed.

In view of the advantages and disadvantages of the above fault diagnosis algorithms, this paper adopts the deep learning method to propose a fault diagnosis model for HVDC transmission lines based on GADF-VGG16. The fault voltage signal of the HVDC transmission line is decomposed into modal components by VMD, and the selected IMF modal components are converted into color images through the Gramian Angular Difference Field (GADF), and the images are input into the improved VGG16 for feature extraction and classification.This method uses a novel GADF encoding method for data preprocessing, and constructs a bijective map for one-dimensional time series and two-dimensional space series, which will not cause the loss of feature information[19].At the same time, this paper improves the traditional VGG16 model structure, increases the BN layer and the convolution layer of the dense connection structure, speeds up the training and convergence speed of the network, and realizes the reuse and enhancement of features.At the same time, the global average pooling layer is used to replace the fully connected layer, which reduces the amount of model parameters and computing time, making the proposed method more suitable for rapid fault diagnosis.The experimental results show that the fault diagnosis model proposed in this paper has high accuracy, is not affected by fault type, transition resistance and fault distance, and has good anti-interference ability and fault tolerance.

The added literature:

1. [13] Zhenzhen Jin, Deqiang He, Zexian Wei,Intelligent fault diagnosis of train axle box bearing based on parameter optimization VMD and improved DBN,Engineering Applications of Artificial Intelligence,Volume 110,2022,104713,ISSN 0952-1976,<https://doi.org/10.1016/j.engappai.2022.104713.>
2. [14] H. Zhao et al., "Intelligent Diagnosis Using Continuous Wavelet Transform and Gauss Convolutional Deep Belief Network," in IEEE Transactions on Reliability, 2022, doi: 10.1109/TR.2022.3180273.
3. [15] Gültekin, Ö., Çinar, E., Özkan, K. et al. A novel deep learning approach for intelligent fault diagnosis applications based on time-frequency images. Neural Comput & Applic 34, 4803–4812 (2022). <https://doi.org/10.1007/s00521-021-06668-2>
4. [16] X. Wang, D. Zhang, X. Zhang and P. Ni, "Fault diagnosis method for hybrid HVDC transmission system based on stacked sparse auto-encoder," 2022 7th Asia Conference on Power and Electrical Engineering (ACPEE), 2022, pp. 1771-1776, doi: 10.1109/ACPEE53904.2022.9783919.
5. [24] TONG yu, PANG xinyu, WEI zihan. Fault diagnosis method of rolling bearing based on GADF-CNN[J]. Vibration and shock, 2021,40(05):247-253+260.
6. [26] Yang, H., Ni, J., Gao, J. et al. A novel method for peanut variety identification and classification by Improved VGG16. Sci Rep 11, 15756 (2021). <https://doi.org/10.1038/s41598-021-95240-y>

Explanation of the added literature:

Reference [13] used VMD to decompose the signal, and extracted the feature information of the modal component with the largest correlation coefficient through the multi-scale distribution entropy. This paper uses the data processing method of [13] to preprocess the fault signal. First, the fault signal is decomposed by VMD to obtain K modal components, and then the correlation coefficient method is used to select the modal component that is most similar to the original signal as the characteristic signal. In the field of bearing fault diagnosis, literature [14], [15], [24] and others proposed a data processing method to convert one-dimensional data into two-dimensional images. The GADF coding method is novel, and the bijective mapping is constructed in one-dimensional time series and two-dimensional space series, which will not cause the loss of feature information. Therefore, this paper adopts GADF encoding method to convert the preprocessed IMF components into two-dimensional color images.The introduction of deep learning has brought new ideas to the field of transmission line fault diagnosis. For example, [16] made full use of the self-learning ability of deep learning and proposed to use DBN to extract and identify fault features. In view of the remarkable achievements of CNN in the field of image classification, this paper draws on the excellent VGG16 model in the literature [26] as the feature extraction model of this paper, and makes a series of improvements to the original VGG16 model to make it more suitable for HVDC transmission lines. fault diagnosis.

6、The reply to comment (6) is as follows：

The author has carefully checked the text for grammatical errors and corrected them to improve the clarity and readability of the text.

Reviewer #3:

Comment :

The authors made the requested modifications and improved their paper.I appreciate the detailed responses given to the reviewers. The authors made an important change in the abstract and this is helpful for the readers. Also the references have been corrected to be more suitable to the research. This is an important research and should be continued.

An explanation of modification:

I am very grateful to the reviewers for their recognition. The authors have made further revisions for the shortcomings of the article. I hope that the reviewers will criticize and correct the revised manuscript submitted by the authors.
